# Targeting STAT3 and STAT5 in Cancer

**DOI:** 10.3390/cancers12082002

**Published:** 2020-07-22

**Authors:** Elvin D. de Araujo, György M. Keserű, Patrick T. Gunning, Richard Moriggl

**Affiliations:** 1 Centre for Medicinal Chemistry, University of Toronto at Mississauga, Mississauga, ON L5L 1C6, Canada; e.dearaujo@mail.utoronto.ca (E.D.d.A.); patrick.gunning@utoronto.ca (P.T.G.); 2 Department of Chemical and Physical Sciences, University of Toronto at Mississauga, Mississauga, ON L5L 1C6, Canada; 3 Medicinal Chemistry, Research Center for Natural Sciences, 1117 Budapest, Hungary; keseru.gyorgy@ttk.mta.hu; 4 Institute of Animal Breeding and Genetics, University of Veterinary Medicine, A-1210 Vienna, Austria

## 1. Introduction

**Figure 1:**
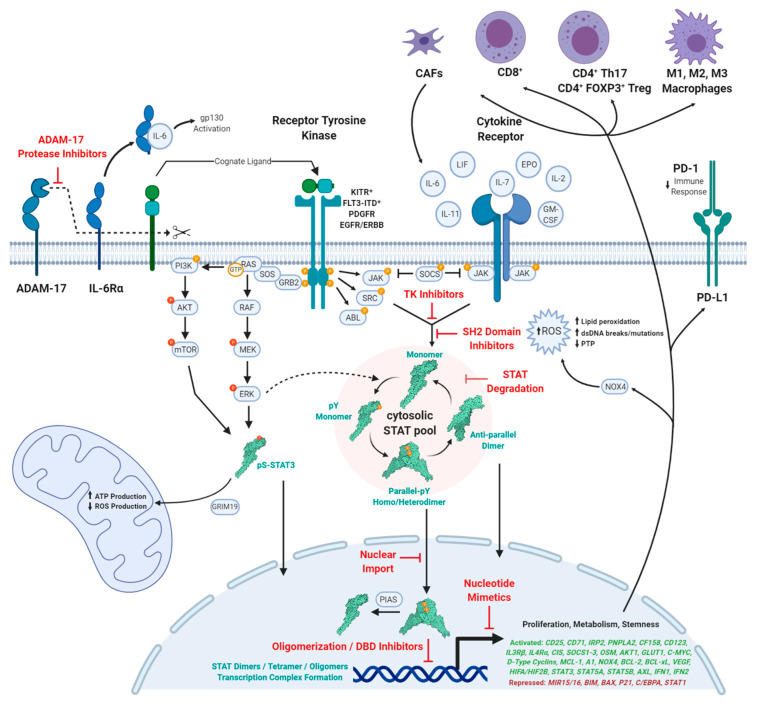
STAT activation pathway with opportunities for inhibition.

Insights into the mutational landscape of the human cancer genome coding regions defined about 140 distinct cancer driver genes in 2013, which approximately doubled to 300 in 2018 following advances in systems cancer biology studies [1,2]. The rapid growth and understanding led to taxonomical organization of known oncogenes into 12 core cancer pathways that regulate cellular differentiation, survival, and genome maintenance [1,3]. Despite significant advances in functional cancer genomics, only a subset of these onco-targets has been successfully tackled in clinical settings, with a predominant emphasis on small molecule kinase inhibitors. Contrastingly, targeting transcription factors has been challenging due to shallow binding sites and non-contiguous surfaces. However, several signaling networks converge upon the Janus Kinase-Signal Transducer and Activator of Transcription (JAK-STAT) pathway and are explored in this special issue (Figure 1). Herein, we describe current concepts and paradigms of oncogenic STAT3/5 targeting. 

The JAK-STAT pathway controls cell survival, differentiation, and metabolism, with critical roles in shaping the chromatin landscape, immune response, and mitochondrial function (Figure 1) [3]. To add another layer of complexity, current STAT studies are focused on in vitro model systems, which may not fully represent the intricate complexities of stimulatory or inhibitory cancer cell-stroma interactions as well as paracrine and endocrine cellular signaling. Additionally, the pleiotropic action of the JAK-STAT pathway in different cell types is complex. It is well known that JAK-STAT3/5 hyperactivation promotes tumorigenesis, both in solid tumors as well as blood cancers. As such, we have focused the themes of this special issue on targeting STAT3 and STAT5, while also highlighting the effects of changes to the collective expression and activity profiles. This is critical as the entire signaling cascade is interconnected with iterative response profiles, best illustrated by the tumor suppressive action of STAT family members, STAT1/2.

The first chapter explores targeting concepts of STAT3/5, with an emphasis on hematopoietic cancers and disease-associated mutations, providing both overview articles as well as original work. Chapter 2 discusses the role of STAT3 and STAT5 across a wide range of solid cancers. Chapter 3 highlights emerging concepts influencing upstream or downstream regulatory mechanisms for targeting oncogenic STAT3/5 action. Each chapter is prefaced with an overview article to appropriately outline and introduce the disease topics for targeting concepts.

An oversimplification of the definition of core cancer pathways is helpful for pharmacologic targeting concepts, since one can aim to target single or multiple core cancer pathways with pathway blocker(s). The JAK-STAT signaling pathway plays a dominant role interconnecting and driving several core cancer networks. Hyperactivated JAK-STAT activity is associated with aberrant functioning of cytokine, growth factor, chemokine, and hormone signaling pathways, ultimately transforming chromatin landscapes and gene reprogramming [3]. Stimulating ligands normally trigger JAK-STAT action in a transient or gradient-like manner. This is well documented for Wnt signaling in crypt villi or upon expansion of the immune system during infection, where a cytokine storm is followed by down-regulation to homeostatic conditions and memory response [4,5,6]. However, the situation in the cancer phenotype is very different and where age dominant clones can originate. For example, 10–15% of individuals over the age of 70 develop Clonal Hematopoiesis of Indeterminate Potential (CHIP), whereby small imbalances of external signals are known to promote acute leukemia and lymphoma outbreak [7,8]. 

Loss of control over cytokine signaling, as observed with gain-of-function mutations in oncogenic JAK-STAT components, directly promotes neoplastic outgrowth [9,10,11]. Moreover, in recent years, advancements in understanding processes, such as unphosphorylated uSTAT action [12] as well as STAT involvement in mitochondrial metabolism [13], have transitioned the consensus away from the idea of a linear signaling JAK-STAT cascade. Several negatively acting controls are also implemented on JAK-STAT signaling to suppress transformation [10,11]. However, cancer is a multi-genetic disease, and lost or lowered tumor suppressor protein function associated with global methylation changes [14] decreased proteolysis of receptor-kinase components (e.g., lost or methylated SOCS family members) [15] and/or inhibited phosphatase action [16] can all provoke hyperactivated JAK-STAT action, culminating into cancer initiation and progression. Within the scope of this special issue, several articles also focused on STAT3/5 hyperactivity in the microenvironment for complex immune cell function or interplay with cancer cells or other stromal components. Overall, aberrant STAT3/5 functioning extends beyond cancer phenotypes into additional malignances, including autoimmunity, chronic inflammation, and infectious disease.

The successful implementation of clinically approved JAK kinase inhibitors (ruxolitinib, tofacitinib, baracitinib, and ifacitinib) validates the therapeutic utility of JAK-STAT targeting in a wide variety of cancers and chronic inflammatory and autoimmune diseases. Current JAK kinase inhibitors are ATP competitive analogues, with limited subtype selectivity. Some selectivity was achieved by covalently targeting a Cys residue in close proximity to the orthosteric pocket of JAK3 [17]. JAK kinase inhibitors have recently been employed for autoimmune-driven diseases including rheumatoid arthritis and exploration for basket trials in cancer, such as those performed for BRAF or HER2 kinase inhibitors, and could further propel clinical use [18]. However, there are significant challenges to overcome in cancer targeting, and several of the proposed inhibitors in literature could be enhanced through deeper understanding of the direct biophysical and target engagement profiles, in conjunction with phenotypic screening. 

Here, we summarize targeting approaches on STAT3/5, where we combine review articles from experts in the field as well as original articles from multiple groups employing different targeting strategies to advance clinical development. Inhibitor designs and medicinal chemistry approaches are covered, highlighting limitations in the current modalities, such as selectivity, potency, and in vivo efficacy for targeting hematopoietic and solid cancers. We have structured this special issue into three main chapters.

**Chapter 1: Targeting STAT3/5 in Hematopoietic Cancers**

This chapter examines the drug targeting concept of STAT3/5 in hematopoietic cancers as well as exploring less-studied STAT-driven indications. The chapter is prefaced by a review outlining a brief history and identification of the involvement of STAT3 and STAT5 gene products and their current mutational landscape. The major gain-of-function driver mutations in hematologic cancers are highlighted, as well as detailed reports of indirect and direct inhibitors of STAT3 and STAT5 and their current stage of clinical development [19]. These themes are further developed by Orlova et al., where the current paradigms in direct small molecule targeting of STAT3 and STAT5 are explored in the context of unphosphorylated STATs, as well as chromatin remodeling [20]. Furthermore, the involvement of STAT signaling in the immune system and T-cell development have also led to proposed studies for hijacking and re-wiring the immune suppressive tumor-associated macrophages in the context of STAT3/5 [21]. The central ideas are expanded upon by Rébé et al., where they examine the critical role of STAT3 in the immune system and T-cell differentiation as well as checkpoint inhibitors [22]. As seen above, several STAT3/5 targeting options were explored for hematopoietic cancers. However, the role of STAT3 and STAT5 in specific indications is still being established and is often revealed through somatic mutations identified from patient tumors. The molecular-level functional effects of specific disease-associated SH2 domain mutations are highlighted in the context of the current protein crystal structures [23]. Additional studies have explored the role of STATs in hematological malignancies. Phospho-STAT5 was shown to play a key role in CD34+/CD38− myeloproliferative neoplasms with downregulation by pharmacologic inhibition of JAK and STAT [24]. JAK/STAT mutations were also identified in patients with the rare and aggressive T-cell prolymphocytic leukemia (T-PLL), pointing towards a possible mode of transformation [25]. Additionally, STAT3 (and not STAT5B) mutations were identified in multiple peripheral T-cell lymphomas (PTCL), as well as high pY-STAT3 expression, particularly in angioimmunoblastic T-cell lymphoma (AITL) and anaplastic large cell lymphoma (ALCL) patient samples, two different PTCL cancer types [26]. From an inhibition perspective, a derivatized indole was shown to be effective in both chronic myeloid leukemia (CML) and acute myeloid leukemia (AML), as well as aggressive STAT5-N642H tumors [27]. Collectively, these studies shed light on the importance of STAT3/5 in several hematopoietic cancers, potentially offering disease biomarkers and hallmarks across several different indications. 

**Chapter 2: Targeting STAT3/5 in Solid Cancers**

This chapter highlights the role of STAT3/5 across a range of solid cancer models and is prefaced by a thorough review highlighting the importance of STAT3 in inflammation, stemness, and mitochondrial functions. It also depicts the mutational landscape with recurrent hotspot mutations in the three different STAT3/5 gene products throughout these solid cancers [28]. The detailed mutational maps, related to the oncogenic STAT3/5 proteins, indicate key functional amino acid residues, and their appearance highlights where they might play critical cell-type specific roles [28]. Successively, Polak et al. highlight the importance of leveraging selective STAT-targeting, arising from the STAT3-driven cancer-stem cell properties and STAT3/5-enhanced immunosuppression, in contrast to the tumor suppression activity of STAT1/2 [29]. These concepts form the foundation for the discussion of STAT3/5 across multiple solid cancers and indications. Notably, aberrant STAT3/5 function alters tumorigenic properties across multiple organs, further highlighting their critical role as cancer drivers. Herein, we briefly outline the different indications explored within the chapter. 

Melanoma and causatively linked autoimmune diseases are directly tied to hyperactivation of STAT3/5 cascades, and dual/simultaneous targeting could reduce the overall disease burden [30]. For colon cancer-based therapies, upstream targeting of IL-6 trans-signalling was explored in the context of the ligand-releasing protease, ADAM17 [31]. Aggressive epithelial ovarian cancer was discussed relative to the STAT3/5 activating tumor microenvironment and their correlation to the persistence of recurrent neoplasms [32]. In prolactin-driven prostate cancer mouse models, STAT5A/B deletions were found to significantly delay the onset of tumorigenesis, further highlighting a potential targeting opportunity [33]. For breast cancer-based models, STAT3 was identified to promote PDL-1 expression, leading to reduced immune responses, which was validated through gene silencing and pharmacologic inhibition [34]. Direct targeting nanomedicine-based approaches were also applied in syngeneic glioblastoma mouse models, where lipid-formulated siRNA was capable of suppressing STAT3 activity and tumor growth [35]. STAT3 activation was also explored in hepatocellular carcinoma (HCC) arising from *hepatitis C* infections. Aydin et al. identified that HCC progression was mediated through NRF2, leading to STAT3 activation and suppression of *miR-122* [36]. Finally, nano-formulated STAT3 inhibitors were also shown to be effective in myeloma xenograft models, enhancing the therapeutic properties of the inhibitor alone [37]. 

Collectively, the range of indications examined in this chapter underscore the importance of STAT3/5 signalling in transformation, but also the potential for cross-indication utility for prospective therapies. 

**Chapter 3: General Targeting Aspects to Block the JAK1/2/3/TYK2-STAT3/5 Core Cancer Pathway**

This chapter broadens the scope of current STAT targeting strategies. This has led to studies of STAT within companion animals, particularly canine species, that offer advantages over current pre-clinical models which are also a major hurdle in drug development safety/toxicity studies. Moreover, companion animals share the same environment as their owners and similarities in cancers, e.g., driver mutation conservation for key STAT domains could be important for understanding the mechanistic detail within comparative pathology studies. Different animal species can also provide additional mechanistic insights into STAT evolution and the signaling cascades [38]. For instance, understanding STAT activation in the context of heat shock proteins (HSPs) is critical for their involvement in cellular stress and the immune response which was explored by Jego et al [39]. 

Alternative strategies for STAT targeting were also discussed within this chapter. Nucleotide therapeutics, which were directed towards the STAT3 DNA-binding domain, were also employed in clinical trials and represent a promising avenue in STAT inhibition that was distinct from small molecules [40]. Alternatively, targeting the specific nucleocytoplasmic STAT transport shuttles offers a creative mechanism for gaining STAT-inhibition selectivity [41]. The role and importance of targeting TYK2, in addition to the JAK1-3, in the context of inducing STAT activation, was also thoroughly discussed [42]. Similarly, the mTOR-STAT3 axis was investigated in Shwachman-Diamond syndrome as a potential kinase target in blockading persistent STAT3 activation [43]. Collectively, these strategies offer non-conventional approaches to the JAK-STAT targeting regimens. 

## 2. Conclusions

In summary, this special issue offers an overview of different STAT3/5 targeting approaches in the context of multiple disease indications. Emerging drug design strategies and medicinal chemistry approaches, including methods to impair function [44] destabilize or degrade transcription factors [45,46,47], interfere with interaction partners and cofactors [48], block DNA binding [49], nuclear shuttling [41], or target specific subsets of cell types/microenvironment [50], initiated new concepts for broad views on targeting. Consequently, targeting oncogenic transcription factors of the STAT family, namely STAT3, STAT5A, and STAT5B as three distinct gene products are major funnels for gene regulatory processes, including chromatin remodeling. Targeting these STAT gene products has therapeutic power, as demonstrated with proof-of-concept studies, although advanced clinical trials are not available. Thus, greater efforts need to be undertaken to identify new targets and develop selective and less toxic clinical-grade inhibitors.

From a contemporary standpoint, the COVID-19 pandemic has highlighted the vulnerability of cancer patients to infection [51]. We want to conclude that research on STAT3/5 targeting molecules and concepts are broadly underexplored, as the 2020 SARS-CoV-2 pandemic revealed. SARS-CoV-2 infection accounts for ≈28% mortality in New York cancer patients, from a cohort of 218 infected individuals. Even therapeutic success will leave potential comorbidities that can flip the coin between life and death [52]. SARS-CoV-2 infection rates were also associated with a 37% higher mortality in hematologic malignancies predominantly associated with CHIP, pointing to the importance of regulation of the blood and immune system, which is under the control of external stimuli, i.e., the JAK-STAT pathway. Currently, mortality of SARS-CoV-2 infections can be reduced by blocking the cytokine storm paradoxically with either tocilizumab or dexamethasone [53,54]. These drugs display well known immunosuppressive action aside from other more systemic impacts on metabolism. STAT3/5 are well known to interact with the glucocorticoid receptor. They can promote cytokine and growth factor feed forward loops, and are critically involved in cytokine or growth factor, as well as hormone signalling. Whether or not STAT3/5 inhibition is beneficial to treatment of COVID-19 infections is unclear, illustrating the gaps in the research ahead of us, as well as the many opportunities, options, and value in targeting STAT3/5.
